# Patient attitudes toward artificial intelligence in Jordanian Healthcare: A cross-sectional survey study

**DOI:** 10.1371/journal.pdig.0001285

**Published:** 2026-07-09

**Authors:** Zeena Al-Dabbas, Laith Khandakji, Nour Al-Shatarat, Hala Alqaisiah, Yazan Ibrahim, Tala Awed, Harith Baik, Mohammad Dawoud, Raghad Al-Haj Ali, Zaid Telfah, Yamamah Al-Hmaid, Adham Alsharkawi

**Affiliations:** 1 Faculty of Medicine, Al-Balqa Applied University, Al-Salt, Jordan; 2 Faculty of Medicine, Jordan University of Science and Technology, Irbid, Jordan; 3 Faculty of Medicine, Yarmouk University, Irbid, Jordan; 4 Department of Mechatronics Engineering, School of Engineering, The University of Jordan, Amman, Jordan; The Chinese University of Hong Kong Faculty of Medicine, HONG KONG

## Abstract

Artificial intelligence (AI) is increasingly integrated into healthcare delivery, yet patient acceptance in resource constrained settings remains incompletely characterized. This study assessed attitudes toward AI supported care among patients attending hospitals in three Jordanian governorates (Amman, Balqa, Irbid) and examined demographic and digital literacy correlates of acceptance. In a cross sectional survey (n = 500 complete questionnaires), participants rated exposure to AI in healthcare and five attitudinal domains, namely perceived usefulness or performance expectancy, trust and transparency, privacy and perceived risks, empathy and human interaction, and readiness or behavioral intention, using 25 items on 5 point Likert scales. Patients expressed conditional optimism: empathy and human interaction was most strongly endorsed (M = 4.33, SD = 0.58), alongside relatively high perceived usefulness (M = 3.97, SD = 0.68), while trust and transparency (M = 3.57, SD = 0.74) and readiness (M = 3.66, SD = 0.90) were moderate to high; privacy and risk concerns were moderate (M = 3.51, SD = 0.77) and self reported exposure was lowest (M = 2.57, SD = 1.07). The highest agreement item indicated preference for AI to work alongside physicians rather than be relied on alone (M = 4.47, SD = 0.81). Trust and transparency and perceived usefulness were positively associated with readiness (r = 0.48 and r = 0.44, respectively; p < .001), while privacy and perceived risks were negatively correlated with trust and usefulness. In multivariable regression adjusting for gender, age group, education, prior AI health app or device use, and self rated digital skill, lower educational attainment and self-rated digital skill were associated with readiness (R^2^ = 0.101). These findings suggest that implementation strategies in Jordan should emphasize human involvement alongside AI, transparent communication and governance, and attention to education- and digital-confidence-related differences in readiness.

## Author summary

AI is increasingly used in healthcare, for example to support diagnosis, triage, and treatment decisions. Whether these tools are accepted by patients depends not only on how well they work, but also on whether patients trust them, understand how they are used, and feel their privacy is protected. Evidence on patient views in middle income and resource constrained settings is still limited. We surveyed 500 patients attending hospitals in three Jordanian governorates to understand how they view AI supported care. Patients generally expected AI to be useful, but they strongly preferred that clinicians remain actively involved and that AI supports rather than replaces physicians. Trust and perceived usefulness were closely linked to willingness to accept AI enabled care, while privacy concerns were present and shaped trust. Readiness to accept AI was lower among participants with lower educational attainment and higher among those with greater self rated digital skill. These findings suggest that successful implementation in Jordan should prioritize transparent communication, strong privacy safeguards, and human centered workflows, while accounting for education- and digital-confidence-related differences in readiness.

## Introduction

AI in health care is increasingly being deployed as clinical tools that support decision-making and care workflows, with a prevailing view that AI should augment clinicians rather than replace professional judgment or accountability [1]. Advances in generative and multimodal foundation models have expanded capabilities while also broadening the risk surface, raising expectations for safety, transparency, privacy protection, and accountability across the system lifecycle [2,3]. Regulators are clarifying lifecycle oversight for AI-enabled software functions that may change after authorization, including predetermined change control plans to support iterative improvement while maintaining reasonable assurance of safety and effectiveness [4].

Credible adoption depends on rigorous clinical evaluation and transparent reporting. DECIDE-AI provides guidance for early-stage clinical studies of AI decision-support systems [5], and newer reporting standards extend requirements to clinical prediction models, diagnostic accuracy studies using AI, and studies involving large language models [6–8]. Because these systems influence patient-related decisions, safe use requires appropriate trust calibration and reliance [9]. A systematic review indicates that patients and the public often anticipate benefits from clinical AI but prefer human oversight and report concerns about transparency and accountability [10]. Evidence of proxy-driven bias in widely used health algorithms shows that equity risks are practical rather than hypothetical, motivating context-specific assessment of benefit, trust, privacy expectations, and effects on the clinician-patient relationship [11].

### Recent literature on attitudes toward AI-supported care

Across settings, the literature converges on a consistent conclusion: stakeholders often view AI in healthcare as promising, but acceptance is typically conditional, shaped by perceived usefulness, trust and transparency, privacy and perceived risks, empathy and human interaction, and readiness or behavioral intention [10,12]. Framing prior evidence around these constructs provides a direct rationale for the attitudinal domains assessed in the present study within Jordan.

Perceived clinical value, especially improved accuracy, efficiency, or timeliness, is a recurrent driver of acceptance. In a large multinational survey of hospital patients across diverse health systems, respondents generally supported AI use but expressed preferences indicating that AI should add value without displacing clinician judgment [13]. Experimental evidence similarly suggests that acceptance increases when AI is presented as more accurate and when recommendations are communicated in ways that emphasize patient centered benefit [14]. In the United States, synthesis of nationally representative surveys indicates that many members of the public expect medicine to benefit from AI, while support varies across applications and population subgroups [12]. At the same time, expectations regarding whether AI will improve affordability and access may be modest and closely tied to broader trust in the healthcare system [15]. Collectively, these findings imply that perceived usefulness is not a generic enthusiasm for technology; it depends on whether AI is understood as improving outcomes patients care about, such as diagnostic reliability, access, and convenience, and whether those benefits are communicated credibly.

Trust emerges as both a direct determinant of acceptance and a mechanism through which other concerns are filtered. Multiple patient studies show that individuals are more comfortable with AI when it is positioned as clinician supervised support rather than autonomous decision making [13,16]. Qualitative work in wound care reinforces that limited AI understanding is common, and that trust often operates through clinician mediation: patients prefer clinicians to interpret and contextualize AI outputs, while transparency and alignment with professional judgment strengthen confidence [17]. From the clinician side, surveys emphasize governance concerns, including accountability, confidentiality, and bias, and support the norm that patients should retain access to human clinical judgment and second opinions [18]. These patterns motivate measuring transparency and trust together; in practice, trust frequently depends on whether AI use is explainable, supervised, and embedded within established clinical responsibility.

Even when general attitudes are positive, privacy and risk concerns remain among the most durable barriers. Public evidence in the United States shows that comfort with AI does not necessarily translate into comfort with data access, and that perceived bias and discrimination risks may meaningfully shape acceptance for some groups [12,19]. Clinician perspectives similarly underscore confidentiality and bias as persistent concerns that require clear regulatory and ethical frameworks [18]. These concerns are directly relevant to measuring perceived risks and privacy attitudes among patients, particularly in contexts where public discussion of data governance and accountability may be limited.

A distinct and repeatedly observed constraint on acceptance is fear of reduced interpersonal care. In sensitive treatment contexts, such as radiotherapy, patients may view AI implementation more negatively when it is perceived to diminish personal interaction or relational support [20]. In the Arab region, student and trainee samples also express concerns that AI lacks empathy and may not adapt flexibly to individualized patient needs [21]. This construct is especially important for patient facing services in which reassurance, communication, and dignity are central to perceived quality of care.

Across stakeholders, enthusiasm often coexists with limited knowledge and limited formal preparation. A systematic review and meta analysis across medical, dental, and nursing student populations found generally positive attitudes alongside only moderate knowledge, implying an enthusiasm preparedness gap that may influence how confidently future clinicians communicate about AI to patients [22]. Similar patterns are reported in specific trainee groups, including radiology residents [23] and nursing students [24]. Because patient acceptance is partly shaped by clinical communication and implementation quality, readiness within the healthcare workforce provides critical context even in patient centered studies.

Arab region studies broadly align with international findings but highlight two recurring themes: strong optimism about AI potential and substantial variability in knowledge and comfort. Medical student surveys in Kuwait and multi country Arab samples report high perceived importance of AI and strong support for AI education, yet also document pronounced knowledge deficits [25,26]. In Lebanon, similar gaps between awareness and deeper understanding have been reported [27]. Patient and public facing data in the region remain comparatively limited and often concentrate on radiology or specific implementations. In Saudi Arabia, patients expressed interest in AI supported radiology but still valued direct interaction with radiologists [28], while public samples demonstrate generally positive orientations tempered by concerns about safety, trust, regulation, and workforce impact [29]. Evidence from the United Arab Emirates also indicates that public perceptions can be mixed and may skew unfavorable in some samples, reinforcing the need for context sensitive measurement rather than assuming uniformly positive attitudes [30]. These regional patterns support measuring both perceived usefulness and relational and ethical concerns, including privacy, empathy, and transparency, when assessing acceptance.

Jordan specific evidence to date has largely emphasized clinicians and students rather than patients, consistently reporting interest in AI alongside barriers related to training, resources, and governance. A survey of healthcare professionals in Jordan documented perceptions of AI alongside perceived barriers and risks [31]. Nursing focused work similarly links AI related knowledge, attitudes, practices, and perceived barriers to workforce outcomes such as intent to stay [32]. Among medical and allied health students, studies commonly report moderate knowledge, strong interest, and calls for curricular integration of AI [33–36]. Specialty focused samples, including radiology and anesthesiology, further illustrate cautious optimism coupled with concerns about role change and human interaction [37,38].

Despite this growing stakeholder literature, direct evidence on patients’ attitudes within Jordan, and critically, how key constructs such as usefulness, trust and transparency, privacy and perceived risks, and empathy and human interaction relate to behavioral intention across demographic and digital literacy groups, remains limited. This gap motivates the present study’s focus on patient attitudes within the Jordanian healthcare system and their relationship to acceptance of AI supported care.

## Materials and methods

### Study design and setting

We conducted a cross-sectional questionnaire survey among a convenience sample of 500 adults recruited from seven tertiary public and academic teaching hospitals across three Jordanian governorates (Amman, Balqa, and Irbid). Data collection took place between July and October 2025.

### Participants and data collection

Eligible participants included adults (aged ≥18 years) attending the participating hospitals who were capable of providing informed consent. The study was approved by the Institutional Review Board (IRB) of Al-Balqa Applied University (Approval No. 2026/2025/2/25). Recruitment was conducted in outpatient clinics, inpatient wards, and waiting areas using a convenience sampling approach. Trained research staff approached potential participants to explain the study’s purpose and obtain informed consent, which was documented by checking a box on the electronic form. The questionnaire was administered in Arabic by the research staff using electronic devices (smartphones or tablets).

Prior to the main study, the questionnaire was pilot tested with 44 participants from the participating hospitals. Feedback from the pilot test and internal consistency analysis were used to refine item wording and improve clarity before final data collection.

## Measures

Demographic and background variables included age group, gender, highest education level, governorate, hospital selected from a standardized drop-down list, prior use of AI-based health applications or devices (Yes/No/Not sure), and self-rated digital skill (Low/Moderate/High).

Attitudes toward AI in healthcare were measured using 25 Likert-type items rated on a 5-point scale (1 = strongly disagree, 2 = disagree, 3 = not sure, 4 = agree, 5 = strongly agree). Items were grouped a priori into six constructs: Exposure to AI in healthcare (2 items), Trust & transparency (5 items), Perceived usefulness / performance expectancy (5 items), Privacy & perceived risks (5 items), Empathy & human interaction (5 items), and Readiness / behavioral intention (3 items).

In this manuscript, trust refers to appropriately calibrated willingness to rely on AI-supported healthcare when its use is transparent, clinically supervised, and embedded within clinician accountability, rather than unconditional confidence in autonomous AI decision-making.

AI exposure was assessed at a general level through items on awareness that AI is used in some hospital departments, previous receipt of AI-assisted examination or care, and prior use of AI-based health applications or devices. The questionnaire did not ask participants to identify specific AI modalities or clinical use cases, such as imaging algorithms, clinical decision-support tools, chatbots, symptom checkers, triage systems, or wearable-device applications.

Composite scores were calculated as the mean of items within each construct. Higher scores indicated greater exposure, higher trust, higher perceived usefulness, greater privacy/risk concern, stronger preference for empathy/human interaction, and higher readiness/behavioral intention, respectively.

### Statistical analysis

Data integrity was assessed by checking value ranges, internal consistency of coding, and completeness of responses. There were no missing item responses, so all analyses were conducted on complete cases. Internal consistency of each construct was evaluated using Cronbach’s alpha (α).

For descriptive statistics, we calculated means (M), standard deviations (SD), and 95% confidence intervals for each item and each construct. Group differences in construct scores were examined using Welch’s *t*-tests for two-group comparisons (e.g., gender, prior AI health app/device use [Yes vs No, excluding “Not sure”]) and one-way analysis of variance (ANOVA) for age group, education level, and self-rated digital skill (Low/Moderate/High), with Tukey post-hoc comparisons where appropriate. Effect sizes were reported as Hedges’ *g* for *t*-tests and $\eta^{2}$ for ANOVA.

Pearson correlation coefficients were computed to quantify associations among the six composite construct scores. Because this involved 15 pairwise inter-construct correlations, Benjamini–Hochberg false discovery rate (FDR) correction was applied to the correlation p-values. To examine independent associations with behavioral intention, we fitted a final adjusted linear regression model with the Readiness or behavioral intention composite as the dependent variable, including education level, prior AI health application or device use, and self-rated digital skill, with gender and age group retained as a priori demographic covariates. Statistical significance was defined as *p* < 0.05 using two sided tests. FDR correction was applied to the inter-construct correlation analyses. Other exploratory group comparisons were not formally adjusted for multiplicity. Analyses were conducted in Python (version 3.12) using the pandas, SciPy, and statsmodels libraries.

## Results

### Data integrity and preparation

The final dataset comprised 500 complete questionnaires with no missing values across 34 variables (9 demographic/administrative fields and 25 Likert-scale attitude items). All Likert-type responses fell within the prespecified range of 1–5, and no out-of-range or implausible values were identified.

### Sample characteristics

Participant characteristics are summarized in Table 1. The sample was 51.6% female (n = 258). The largest age group was < 25 years (26.8%), and the most common highest education level was a Bachelor’s degree (45.4%). Most participants were recruited from hospitals in Amman (45.6%), followed by Balqa (31.8%) and Irbid (22.6%). Just under half of respondents reported no prior use of AI-based health applications/devices (47.8%), and the majority rated their digital skill as moderate (60.2%).

**Table 1:** Participant demographics (N = 500).

Variable	Category	n	%
Age group	<25	134	26.8
Age group	25–34	105	21.0
Age group	35–44	95	19.0
Age group	45–54	74	14.8
Age group	55–64	68	13.6
Age group	≥65	24	4.8
Gender	Female	258	51.6
Gender	Male	242	48.4
Education	Bachelor’s	227	45.4
Education	High school	118	23.6
Education	Diploma/College	75	15.0
Education	<High school	32	6.4
Education	Master’s	25	5.0
Education	Doctorate	23	4.6
Governorate	Amman	228	45.6
Governorate	Balqa	159	31.8
Governorate	Irbid	113	22.6
Hospital	Al-Hussein Al-Salt New Hospital	192	38.4
Hospital	Jordan University Hospital	124	24.8
Hospital	King Abdullah University Hospital	65	13.0
Hospital	Princess Basma Teaching Hospital	50	10.0
Hospital	Prince Hamzah Hospital	50	10.0
Hospital	Al-Hussein Medical City	17	3.4
Hospital	Al-Salt Hospital	2	0.4
Prior AI health app/device use	No	239	47.8
Prior AI health app/device use	Yes	215	43.0
Prior AI health app/device use	Not sure	46	9.2
Self-rated digital skill	Moderate	301	60.2
Self-rated digital skill	High	136	27.2
Self-rated digital skill	Low	63	12.6

### Reliability (internal consistency)

Internal consistency estimates for the six attitude constructs are shown in Table 2. Trust & transparency (α=0.745) and Perceived usefulness / performance expectancy (α=0.756) demonstrated acceptable internal consistency, indicating reasonably coherent measurement of these domains. The remaining constructs showed more modest reliability (Exposure α=0.596; Privacy & perceived risks α=0.665; Empathy & human interaction α=0.637; Readiness / behavioral intention α=0.621), suggesting greater heterogeneity in responses. Findings involving these constructs should therefore be interpreted as exploratory construct-level associations rather than highly precise estimates of distinct latent domains.

**Table 2:** Internal consistency reliability (Cronbach’s alpha) by construct.

Construct	Items (*k*)	Cronbach’s α
Exposure to AI in healthcare	2	0.596
Trust & transparency	5	0.745
Perceived usefulness / performance expectancy	5	0.756
Privacy & perceived risks	5	0.665
Empathy & human interaction	5	0.637
Readiness / behavioral intention	3	0.621

### Construct-level descriptive statistics

**Fig 1:**
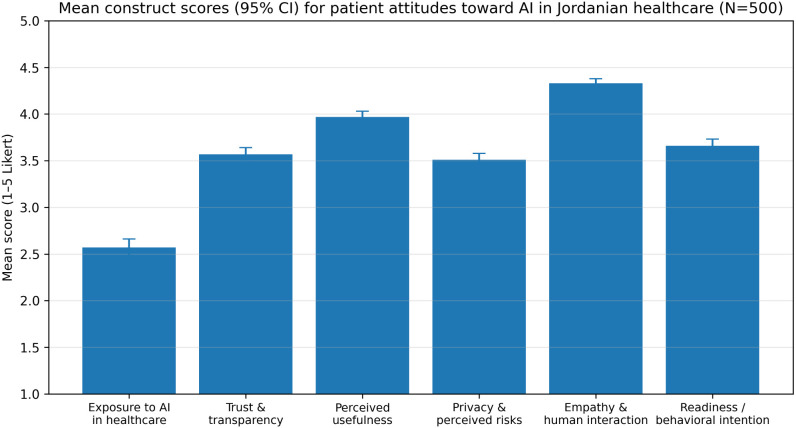
Mean construct scores (with 95% confidence intervals) for patient attitudes toward AI in Jordanian healthcare. Scores range from 1 (strongly disagree) to 5 (strongly agree).

Construct-level descriptive statistics are presented in Table 3 and illustrated in Fig 1. Empathy & human interaction had the highest mean score (M = 4.33, SD = 0.58), followed by Perceived usefulness / performance expectancy (M = 3.97, SD = 0.68). Readiness / behavioral intention had a mean of 3.66 (SD = 0.90), Trust & transparency had a mean of 3.57 (SD = 0.74), and Privacy & perceived risks had a mean of 3.51 (SD = 0.77). Exposure to AI in healthcare had the lowest mean score (M = 2.57, SD = 1.07).

**Table 3:** Construct-level descriptive statistics (1–5 Likert).

Construct	M	SD	95% CI LL	95% CI UL
Exposure to AI in healthcare	2.57	1.07	2.48	2.66
Trust & transparency	3.57	0.74	3.51	3.64
Perceived usefulness / performance expectancy	3.97	0.68	3.92	4.03
Privacy & perceived risks	3.51	0.77	3.44	3.58
Empathy & human interaction	4.33	0.58	4.28	4.38
Readiness / behavioral intention	3.66	0.90	3.58	3.73

### Item-level descriptive statistics

Item-level descriptive statistics are provided in Table 4. The highest mean was observed for, “I prefer AI to work alongside the physician rather than relying on it alone” (M = 4.47, SD = 0.81). Other high-scoring items included human empathy remaining important and preference for a human explanation of test results. The lowest mean was observed for, “Previously received AI-assisted examination/care” (M = 2.29, SD = 1.24).

**Table 4:** Item-level descriptive statistics ranked from highest to lowest agreement. Construct codes: EXP = Exposure; TR = Trust/Transparency; PU = Perceived usefulness; PR = Privacy/Risk; HI = Human interaction; BI = Behavioral intention.

Item	Construct	Item (English)	M	SD
HI5	HI	I prefer AI to work alongside the physician rather than relying on it alone.	4.47	0.81
HI1	HI	Human empathy remains important to me even with AI-based diagnosis.	4.39	0.89
HI4	HI	I feel reassured when a human explains my test results rather than a device.	4.38	0.90
HI3	HI	Heavy reliance on AI may reduce human connection between doctors and patients.	4.25	0.93
BI2	BI	I prefer a shared approach where AI and the physician jointly review my case.	4.22	0.85
PU2	PU	AI can speed up delivery of medical care for patients like me.	4.17	0.84
HI2	HI	AI cannot understand the emotional aspects of healthcare.	4.17	1.02
PU1	PU	AI can help physicians detect diseases early.	4.11	0.87
PU5	PU	AI diagnosis would allow physicians more time for complex tasks.	4.03	0.93
PU4	PU	AI can improve the quality of care I receive.	3.85	0.98
TR5	TR	Would feel comfortable relying on AI if recommended by my physician.	3.85	0.98
PR3	PR	Errors in AI diagnosis could lead to serious health risks for patients.	3.81	1.00
PR5	PR	Concerned AI recommendations could override the treating physician’s judgment.	3.75	1.03
TR3	TR	Trust AI recommendations more when AI/doctor explains reasons for them.	3.72	1.08
PU3	PU	Using AI contributes to lowering treatment costs.	3.70	1.05
TR1	TR	Trust healthcare providers will use AI responsibly.	3.65	1.03
TR4	TR	Trust AI systems in this hospital will not cause me harm.	3.49	1.07
PR4	PR	Difficult to correct mistakes if AI makes a wrong decision about my health.	3.47	1.10
BI3	BI	If AI proven more accurate than doctors, I would accept an AI diagnosis.	3.41	1.35
TR2	TR	Believe AI systems used here are accurate in diagnosing diseases.	3.35	1.06
BI1	BI	If AI as accurate as doctor, I would accept it conducting initial assessment.	3.34	1.30
PR2	PR	Fear personal data may be shared without consent through AI systems.	3.33	1.30
PR1	PR	Concerned my medical data may not be secure if used by AI.	3.18	1.31
EXP2	EXP	Aware that AI is currently used in some hospital departments.	2.85	1.30
EXP1	EXP	Previously received AI-assisted examination/care.	2.29	1.24

### Bivariate group differences in composite scores

Statistically significant bivariate group differences in composite construct scores are summarized in Table 5. Gender was associated with Exposure and Perceived usefulness. Age group was associated with Exposure, Trust & transparency, and Empathy & human interaction. Education level was associated with Exposure, Trust & transparency, Perceived usefulness, Privacy/Risk, and Readiness / behavioral intention. Prior AI health app/device use was associated with Exposure, Trust & transparency, Perceived usefulness, and Readiness / behavioral intention. Self-rated digital skill was associated with Exposure, Trust & transparency, Perceived usefulness, and Readiness / behavioral intention.

**Table 5:** Bivariate associations between participant characteristics and composite construct scores. Only statistically significant associations are shown.

Characteristic	Construct	Test result and effect size	Direction / note
Gender	Exposure	*p* = .031; Hedges’ *g* = 0.19	Female > Male
Gender	Usefulness	*p* = .027; Hedges’ g=−0.20	Male > Female
Age group	Exposure	*F*(5,494) = 2.62, *p* = .024; $\eta^{2}=0.026$	≥65 <<25
Age group	Trust & transparency	*F*(5,494) = 3.10, *p* = .009; $\eta^{2}=0.030$	≥65 <<25
Age group	Empathy & human interaction	*F*(5,494) = 2.35, *p* = .040; $\eta^{2}=0.023$	55–64 > 35–44
Education	Exposure	*F*(5,494) = 9.38, *p* < .001; $\eta^{2}=0.087$	Lower education generally lower
Education	Trust & transparency	*F*(5,494) = 2.44, *p* = .033; $\eta^{2}=0.024$	Small omnibus difference
Education	Usefulness	*F*(5,494) = 2.86, *p* = .015; $\eta^{2}=0.028$	Small omnibus difference
Education	Privacy/Risk	*F*(5,494) = 2.55, *p* = .027; $\eta^{2}=0.025$	No Tukey-adjusted pairwise difference
Education	Readiness	*F*(5,494) = 6.88, *p* < .001; $\eta^{2}=0.065$	Lower education generally lower
Prior AI health app/device use	Exposure	*p*<.001; Cohen’s *d* = 0.40	Yes > No
Prior AI health app/device use	Trust & transparency	*p*<.001; Cohen’s *d* = = 0.38	Yes > No
Prior AI health app/device use	Usefulness	*p* = .002; Cohen’s *d* = 0.29	Yes > No
Prior AI health app/device use	Readiness	*p* = .002; Cohen’s *d* = 0.30	Yes > No
Self-rated digital skill	Exposure	*F*(2,497) = 11.97, *p* < .001; $\eta^{2}=0.046$	Higher skill generally higher
Self-rated digital skill	Trust & transparency	*F*(2,497) = 14.68, *p* < .001; $\eta^{2}=0.056$	Higher skill generally higher
Self-rated digital skill	Usefulness	*F*(2,497) = 11.03, *p* < .001; $\eta^{2}=0.042$	Higher skill generally higher
Self-rated digital skill	Readiness	*F*(2,497) = 15.11, *p* < .001; $\eta^{2}=0.057$	Higher skill generally higher

### Inter-construct correlations

Inter-scale correlations among the six composite constructs are reported in Table 6. Trust & transparency and Perceived usefulness were moderately and positively correlated (Trust–Usefulness: *r* = 0.55, 95% CI [0.48, 0.61]). Both constructs were also positively correlated with Readiness / behavioral intention (Trust–Readiness: *r* = 0.48, 95% CI [0.41, 0.55]; Usefulness–Readiness: *r* = 0.44, 95% CI [0.37, 0.51]). Privacy & perceived risks was negatively correlated with Trust (Trust–Privacy/Risk: r=−0.23, 95% CI [−0.31, −0.15]) and with Perceived usefulness (Usefulness–Privacy/Risk: r=−0.17, 95% CI [−0.25, −0.08]). Privacy/Risk was positively associated with Empathy & human interaction (Privacy/Risk–Empathy/Human interaction: *r* = 0.18, 95% CI [0.10, 0.27]). Exposure to AI in healthcare was positively correlated with Trust (Exposure–Trust: *r* = 0.25, 95% CI [0.17, 0.33]) and with Readiness (Exposure–Readiness: *r* = 0.24, 95% CI [0.16, 0.32]). After FDR correction across the 15 pairwise inter-construct correlations, the emphasized associations remained statistically significant, with FDR-adjusted *p* < .001 for each.

**Table 6:** Inter-scale correlations among composite construct scores, with Benjamini–Hochberg FDR correction across 15 pairwise comparisons.

Pair	*r*	95% CI	Raw *p*	FDR-adjusted *p*
Exposure – Trust	0.25	[0.17, 0.33]	<.001	<.001
Exposure – Usefulness	0.09	[-0.001, 0.17]	.053	.088
Exposure – Privacy/Risk	0.05	[-0.03, 0.14]	.227	.284
Exposure – Empathy/Human interaction	-0.07	[-0.16, 0.02]	.109	.149
Exposure – Readiness	0.24	[0.16, 0.32]	<.001	<.001
Trust – Usefulness	0.55	[0.48, 0.61]	<.001	<.001
Trust – Privacy/Risk	-0.23	[-0.31, -0.15]	<.001	<.001
Trust – Empathy/Human interaction	-0.02	[-0.10, 0.07]	.717	.717
Trust – Readiness	0.48	[0.41, 0.55]	<.001	<.001
Usefulness – Privacy/Risk	-0.17	[-0.25, -0.08]	<.001	<.001
Usefulness – Empathy/Human interaction	0.03	[-0.05, 0.12]	.460	.493
Usefulness – Readiness	0.44	[0.37, 0.51]	<.001	<.001
Privacy/Risk – Empathy/Human interaction	0.18	[0.10, 0.27]	<.001	<.001
Privacy/Risk – Readiness	-0.08	[-0.16, 0.01]	.085	.128
Empathy/Human interaction – Readiness	0.04	[-0.04, 0.13]	.334	.385

### Regression predictors of behavioral intention

A final adjusted linear regression model was fitted with the Readiness / behavioral intention composite as the dependent variable, including education level, prior AI health app/device use, and self-rated digital skill, with gender and age group retained as a priori demographic covariates (Table 7). The overall model was statistically significant (*F*(14,485) = 3.89, *p* < .001) and accounted for approximately 10.1% of the variance in readiness (*R*^2^ = 0.101).

**Table 7:** Multiple linear regression predicting behavioral intention (Readiness/ Intention composite). Model: *F*(14,485) = 3.89, *p* < .001, *R*^2^ = .101.

Predictor	B	SE	*t*	*p*	95% CI
Intercept	3.245	0.199	16.30	<.001	[2.854, 3.637]
Male (vs Female)	0.113	0.080	1.42	.158	[-0.044, 0.270]
Age < 25 (vs 25–34)	-0.025	0.115	-0.22	.825	[-0.251, 0.201]
Age 35–44 (vs 25–34)	-0.110	0.125	-0.88	.379	[-0.355, 0.135]
Age 45–54 (vs 25–34)	-0.014	0.137	-0.10	.920	[-0.284, 0.256]
Age 55–64 (vs 25–34)	0.005	0.141	0.04	.971	[-0.272, 0.282]
Age ≥65 (vs 25–34)	-0.083	0.205	-0.40	.686	[-0.485, 0.319]
Education <High school (vs Bachelor’s)	-0.512	0.171	-2.99	.003	[-0.849, -0.175]
Education High school (vs Bachelor’s)	-0.254	0.103	-2.47	.014	[-0.456, -0.052]
Education Diploma/ College (vs Bachelor’s)	-0.189	0.122	-1.56	.120	[-0.428, 0.050]
Education Master’s (vs Bachelor’s)	-0.198	0.195	-1.02	.309	[-0.581, 0.184]
Education Doctorate (vs Bachelor’s)	0.326	0.203	1.60	.110	[-0.074, 0.725]
Prior AI use: Yes (vs No)	0.105	0.089	1.17	.241	[-0.071, 0.280]
Prior AI use: Not sure (vs No)	-0.068	0.143	-0.48	.634	[-0.348, 0.212]
Digital skill (1 = Low to 3 = High)	0.217	0.072	3.02	.003	[0.076, 0.358]

Compared with participants holding a Bachelor’s degree, those with <High school education (B = −0.512, SE = 0.171, *p* = .003) and those with High school education (B = −0.254, SE = 0.103, *p* = .014) had significantly lower readiness scores, adjusting for other covariates. Self-rated digital skill was positively associated with readiness (B = 0.217 per one-unit increase on the 1–3 scale, SE = 0.072, *p* = .003), indicating higher readiness among participants with greater digital confidence. In contrast, gender, age group, and prior AI health app/device use were not statistically significant predictors in the multivariable model (all *p* > .05).

## Discussion

In this Jordanian hospital-based sample, patients expressed conditional optimism toward AI-supported healthcare. The overall pattern of responses showed favorable perceptions of usefulness and moderate readiness, alongside strong emphasis on empathy, human interaction, and clinician involvement. Privacy and perceived-risk concerns were also present, while self-reported exposure to AI in healthcare was comparatively low. Together, these findings suggest that acceptability was greatest when AI was viewed as clinician-supportive rather than autonomous.

Beyond mean levels, the pattern of associations between constructs helps clarify how acceptance is structured. Trust & transparency and Perceived usefulness were moderately and positively correlated, and both were positively associated with Readiness / behavioral intention (Table 6). These associations suggest that, within this sample, willingness to engage with AI-enabled care was closely linked to perceived benefit and appropriately calibrated willingness to rely on AI-supported care under transparent and clinically supervised conditions. This pattern is consistent with the Technology Acceptance Model, which identifies perceived usefulness and perceived ease of use as central determinants of technology acceptance [39]. In the present healthcare-AI context, the associations involving trust, privacy/risk, and readiness extend this framing by highlighting reliance, perceived security, and governance concerns as additional implementation-relevant dimensions. Privacy & perceived risks were inversely associated with both trust and perceived usefulness, indicating that concerns about harm and data security are central to how patients evaluate AI. At the same time, privacy and risk concerns were only moderate on average, and the combination of moderate concern with moderate or high readiness suggests that worries about privacy do not necessarily prevent willingness to use AI when patients also perceive clear benefits and trust the systems and clinicians involved.

Privacy and perceived-risk concerns require specific interpretation. A moderate score does not indicate absence of concern; rather, it suggests that patients recognized meaningful risks while still perceiving potential benefits from AI-supported care. In this context, relevant risks include unauthorized access to identifiable health information, data breaches or ransomware affecting hospital information systems, insecure transfer of data between hospitals, vendors, cloud services, or mobile health applications, secondary use of patient data for model development without clear notice or consent, limited auditability of data access, and uncertainty about accountability when AI outputs influence clinical decisions. In public-sector or academic hospital settings, patients may also worry about non-clinical or governmental access to health data. However, because the present survey did not directly measure government surveillance or institutional data-access concerns, we discuss this as a contextual privacy-governance issue requiring future research, not as an observed finding. These concerns support the need for transparent data governance, role-based access control, encryption, audit trails, vendor oversight, incident-response capacity, and clear patient-facing communication about how health data are stored, shared, and protected [2].

These implementation priorities align with established cybersecurity and health-IT governance frameworks. The NIST Cybersecurity Framework 2.0 organizes cybersecurity risk management around governance, identification, protection, detection, response, and recovery. The HHS 405(d) Health Industry Cybersecurity Practices guidance provides healthcare-sector practices for managing cyber threats and protecting patients. In this context, the moderate Privacy and Perceived Risk score may reflect patient concern about whether cybersecurity safeguards are visible, reliable, and consistently implemented in AI-enabled healthcare workflows [40,41].

The findings also suggest that education and self-rated digital confidence may be more informative for implementation planning than demographic profiling alone. Age- and gender-related differences were small, whereas lower educational attainment and lower self-rated digital confidence were more consistently associated with lower readiness. These associations should be interpreted cautiously because the study was cross-sectional and self-rated digital skill may reflect perceived confidence rather than objectively measured ability.

Prior use of AI-based health applications or devices was also associated with more favorable attitudinal profiles. Compared with those reporting no prior use, participants with prior use reported higher exposure, trust, perceived usefulness, and readiness, with small to small–moderate effect sizes (Table 5). Taken together, these patterns suggest that prior AI-related experience and self-rated digital confidence were associated with greater readiness to accept AI-enabled care, without clear evidence of reduced concern for privacy or diminished emphasis on human interaction. From an implementation perspective, these associations suggest that self-rated digital confidence and prior AI-related experience should be considered when designing patient-facing AI implementation strategies, rather than being interpreted as evidence that digital-literacy interventions would cause higher readiness.

Psychometric findings provide additional nuance relevant to measurement and to how acceptability is conceptualized. Trust & transparency and Perceived usefulness showed acceptable internal consistency, supporting their use as distinct constructs in this context. By contrast, Exposure, Privacy & perceived risks, Empathy & human interaction, and Readiness / behavioral intention demonstrated more modest reliability (Table 2), which likely attenuates observed associations and suggests that these domains are more heterogeneous. Item level diagnostics (Table 8) indicated that items explicitly addressing division of labor and authority, such as preferring AI to work alongside rather than replace physicians, concern that AI recommendations could override physician judgment, and preference for joint AI and physician review, were less coherent within their assigned subscales than neighboring items. Conceptually, this pattern is consistent with human oversight or shared control forming a partially distinct dimension of AI acceptability that cuts across empathy, perceived risk, and intention. Future work should refine and validate instruments in Arabic speaking settings to capture this oversight and shared control dimension explicitly and to clarify how it interacts with trust, perceived usefulness, and readiness in shaping patient acceptance of AI enabled care.

**Table 8:** Item diagnostic summary for items reflecting oversight and shared control.

Item code and English paraphrase	Corrected $r_{it}$	α if deleted
PR5: privacy or risk, concern that AI recommendations override physician judgment	0.24	0.685
HI5: empathy or human interaction, prefer AI to work alongside the physician rather than alone	0.17	0.675
BI2: readiness, prefer joint review of my case by AI and the physician	0.26	0.714

### Limitations

This study has several limitations. First, the cross sectional design precludes causal inference and all reported associations are correlational. Second, recruitment from selected hospitals in three governorates using non probability sampling limits generalizability to the wider Jordanian population and to other healthcare settings.

Third, all data were based on self report collected through interviewer administered questionnaires, including the digital skill variable, which measured perceived digital confidence rather than objectively tested ability. Responses may therefore be affected by social desirability bias, interviewer effects, subjective misclassification of digital ability, and heterogeneous interpretations of AI (for example chatbots, decision support tools, and imaging algorithms). The exposure items did not distinguish between specific AI modalities or use cases, such as chatbots, clinical decision-support tools, imaging algorithms, triage systems, or wearable-device applications. Because exposure was low, readiness findings should be interpreted as attitudes toward AI-supported care in general rather than as acceptance of a specific AI technology. Because AI modality or clinical use case was not measured, it could not be included as a covariate or potential confounder in the adjusted regression model. Fourth, several constructs showed only modest internal consistency, and item level diagnostics suggested that oversight and authority related items may represent a partially distinct latent dimension that is not fully captured by the current subscales. Formal psychometric work, including factor analysis and test and retest reliability in Arabic speaking populations, is needed before these scales are used for high stakes comparisons.

Fifth, the regression model explained a small proportion of variance in readiness ($R^{2}\approx 0.10$) and included only demographic and technology-related predictors; other potentially important determinants, including detailed facets of trust, perceived fairness, prior healthcare experiences, and specific AI use cases, were not assessed, so residual confounding remains possible. The Privacy and Perceived Risk construct captured general concern about privacy, harm, and data security, but did not separately measure specific risks such as ransomware, third-party vendor access, secondary data use, cross-institutional data sharing, or governmental access to health information. Finally, although FDR correction was applied to the inter-construct correlation analyses, other exploratory group comparisons were not formally adjusted for multiplicity; therefore, statistically significant findings with small effect sizes should be interpreted cautiously.

## Conclusion

In this hospital-based sample from Jordan, patients reported favorable perceptions of AI usefulness, moderate-to-high trust and readiness, strong preferences for empathic clinician involvement, moderate privacy/risk concerns, and limited self-reported exposure to AI in clinical care. Inter-construct correlations showed that trust and perceived usefulness were positively associated with readiness, while privacy/risk concerns were inversely associated with trust and perceived usefulness. Educational attainment and self-rated digital skill were also associated with readiness, but these cross-sectional associations should not be interpreted as evidence that changing digital confidence would cause changes in readiness.

For health system modernization in Jordan and similar settings, these findings support implementing AI primarily as clinician-supportive technology, coupled with clear communication about AI use, explicit privacy and cybersecurity safeguards, transparent data governance, and institutional accountability for data access, data sharing, and incident response. Future research should refine and validate measures of AI acceptability, collect AI use-case-specific exposure data, explicitly assess oversight and shared-control preferences, and examine how evolving experience, policy, and technology influence patient attitudes over time.
